# Effects of Climate Change and Heterogeneity of Local Climates on the Development of Malaria Parasite (*Plasmodium vivax*) in Moscow Megacity Region

**DOI:** 10.3390/ijerph16050694

**Published:** 2019-02-26

**Authors:** Varvara Mironova, Natalia Shartova, Andrei Beljaev, Mikhail Varentsov, Mikhail Grishchenko

**Affiliations:** 1 Faculty of Geography, Lomonosov Moscow State University, Moscow 119991, Russia; mironova.va@gmail.com (V.M.); mvar91@gmail.com (M.V.); m.gri@geogr.msu.ru (M.G.); 2 WHO Consultant on malaria, Former WHO Advisor on malaria, WHO EMRO, Cairo 11371, Egypt; beljaev.moscow@gmail.com; 3 A.M. Obukhov Institute of Atmospheric Physics, 3 Pyzhyovskiy Pereulok, Moscow 119017, Russia; 4 Research Computing Center, Lomonosov Moscow State University, Moscow 119991, Russia

**Keywords:** vivax malaria, urban heat island, climate change, malaria season, *Plasmodium vivax*, Russia, Moscow

## Abstract

The article presents the results of a spatio-temporal analysis of the changes of the favorability of climatic conditions for the transmission of vivax malaria in the Moscow megacity and its surroundings during the period from 1977 to 2016. Using the historical temperature records at urban and rural weather stations, we calculated the key indicators of climate favorability for malaria transmission, *viz*. the sum of effective temperatures, the duration of the season of effective infectiveness, and a new integral index of climate favorability. We demonstrated a dramatic increase of all three indicators, which accelerated after 1984, and a high spatial heterogeneity among them. Due to the urban heat island effect, the degree of climatic favorability is especially high in the densely urbanized areas of Moscow megacity compared with the suburban and rural areas. Climatic conditions for vivax malaria in Moscow are better now than before. The season of effective infectiveness continues in the central part of the city for 25 days longer, and the integral index of climate favorability is 85% higher in comparison to mean values over the rural surroundings. The study contains an alert regarding the risk of malaria resurgence in the Moscow region in the case of the sufficient importation of cases from abroad.

## 1. Introduction

Human malaria is a group of four infections caused by protozoan parasites of the genus *Plasmodium* and transmitted by anopheline mosquitoes. On a global scale, practically all cases of malaria are caused by either *P. falciparum* or *P. vivax*. The former is the predominant of the two (*ca* 90%). The share of infections caused by human parasites *P. malariae* and *P. ovale* is negligible. Humans occasionally become infected with *Plasmodium* species that normally infect animals, such as *P. knowlesi*. As of yet, there are no reports of human–mosquito–human transmission of such zoonotic species of malaria [1]. Particular parasite species have their specific strategies for survival [2], as well as their own features of pathology, epidemiology, public health significance, and amenability to control.

Malaria reintroduction in the territories that had been freed from it is one of the critical issues of malaria control in the 21st century [3]. In the pre-elimination era, malaria was endemic in most of the European countries, including a considerable part of Russia. In Europe, all of the species of malaria ever present (that is *P. malariae, P. falciparum*, and *P. vivax*) had been eliminated in the mid-20th century, and vivax malaria was the last one to disappear. Since then, short-living episodes of the autochthonous transmission of *P. vivax* have been documented in a number of European countries, and, among them, Russia was the most affected.

**Figure 1:**
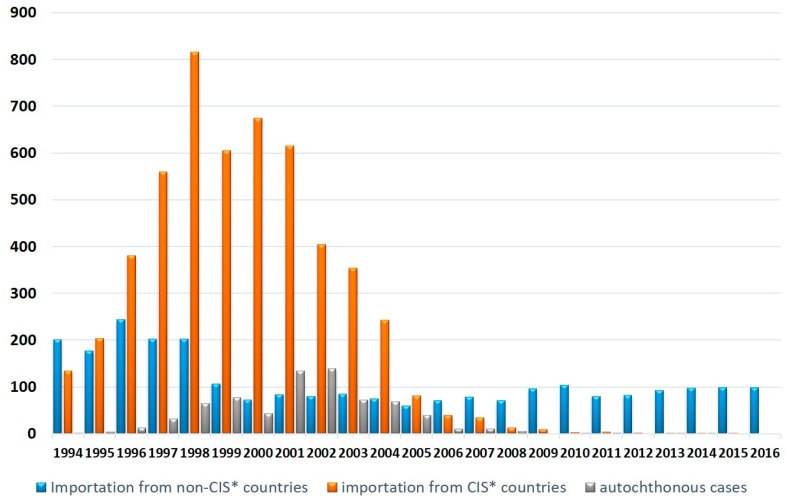
Malaria cases in Russia. Source: Russian Federal Service for Surveillance on Consumer Rights Protection and Human Wellbeing (Rospotrebnadzor). * CIS—Commonwealth of Independent States, consisting of a part of former Soviet Republics.

Between 1979 and 1990, episodes of local transmission of *P. vivax* in Russia occurred mostly due to importation from Afghanistan by the returning Soviet soldiers [4]. Importation of malaria became a problem again after the dissolution of the Union of the Soviet Socialist republics (USSR), which triggered epidemics of vivax malaria in a number of former member states, including Tajikistan and Azerbaijan in particular. During the post-Soviet epoch, the local transmission of malaria in Russia followed ups and downs of malaria in Asiatic ex-members of USSR, with a lag of about two years [5]. Since the dissolution of the USSR in 1991, there were no autochthonous cases of malaria in Russia until 1996. An upsurge of malaria in post-Soviet states provoked secondary transmission in Russia, which happened due to the importation of malaria by migrants and often coincided with hot summer weather. From 1996 to 2009, there was a wave of transmission with more than 700 autochthonous cases. After that, only five episodic autochthonous cases occurred within Russia (Figure 1).

The reintroduction of vivax malaria remains possible in temperate regions; this is why the question of determinants and drivers of malaria re-emergence does not lose its relevance and requires new approaches.

Malaria is often referred to as a climate-dependent disease, which is primarily because a certain sum of temperatures is needed for the complete maturation of sporozoites in mosquitoes. *Plasmodium vivax* requires lower temperatures for its development in the vector than other human malaria species. At temperatures between 16 °C and 18 °C, *P. vivax* would develop, albeit slowly, whereas the development of *P. falciparum* is possible only at 18 °C and above. *P. falciparum* used to be widespread in Europe, including the southernmost areas of Russia. In warm years, it could spread in Russia even up to 61° N [6]. However, the Palaearctic variety of *P. falciparum* had been probably eliminated throughout Europe by the end of the 1950s. The importation of *P. falciparum* from elsewhere is arguably impossible, because the mosquitoes of the Palaearctic region are insusceptible to *P. falciparum* varieties from the Afrotropical and Oriental regions [7,8,9]. Therefore, we discuss here the climatic conditions favoring the possible reintroduction of *P. vivax* only, and leave the question of *P. falciparum* beyond the scope of this work.

Malaria vectors are much more resistant to low temperatures than malaria parasites. Temperature affects the bionomics of *Anopheles* mosquitoes, such as the speed of development of the aquatic stages (which depends on the temperature of the place of breeding), the speed of blood digestion (which depends on the temperature of the resting place), and their survival in general [10]. However, the minimum temperature requirements of malaria parasites during the extrinsic part of the cycle are significantly higher than those of the mosquitoes. For example, aquatic stages of the principal malaria vector in the Palaearctic region, *A. maculipennis s.l*, require no less than +10 °C, and adults actively feed at this temperature, whereas stages of *P. vivax* in mosquitoes die out at temperatures below 16 °C. This was the explanation of the phenomenon of “anophelism without malaria” that puzzled European malariologists in the 1920s and 1930s [11,12].

Hence, in temperate regions, by the time of the year when the temperatures become suitable for the development of the parasite in the mosquito, active vectors are already present in ample quantities. Therefore, we focus on the temperature requirements of parasites, not the vectors, as only the former present the limiting factor.

Malaria transmission occurs both in rural and urban areas, although the rural environment is, as a rule (but not always), more favorable for malaria [13]. The reasons for this are not the same in the tropics and in temperate/subtropical areas. Generally, conditions for urban malaria are less favorable due to the paucity of suitable breeding places and widespread pollution, to which anophelines are much more sensitive than other mosquitoes. On the other hand, the urban areas are usually significantly warmer (by up to 2 to 3 °C) than their rural surroundings due to specific land-cover modifications and anthropogenic factors. Such a temperature anomaly, which is known as an urban heat island effect [14,15], may favor the transmission of malaria. The magnitude of the urban heat islands is comparable with the magnitude of the observed climate changes. The latter allows suggesting the significant effect of the urban temperature anomaly on the climate favorability for malaria transmission.

In temperate and subtropical climates, malaria transmission in large cities is not an exception. In recent decades, it has been repeatedly documented in New York [16], Houston [17], etc. As for Russia, there was an outbreak in Nizhny Novgorod in 1998 [18], Perm between 1998 and 2003 [19], Moscow between 1972 and 1973 and in 1981, and the largest outbreak was between 1999 and 2009 [20].

**Figure 2:**
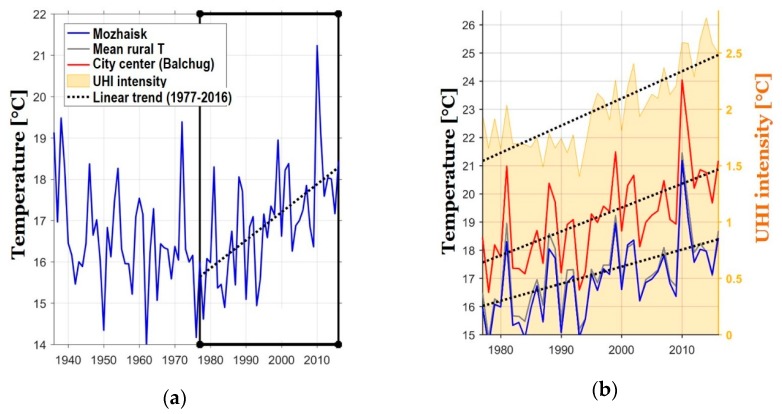
Dynamics of the average summer air temperature at the Mozhaisk station between 1936 and 2016. (**a**); the dynamics of air temperature at Mozhaisk and Balchug stations and the average background temperature (averaged over the Naro-Fominsk, New Jerusalem, Klin, Dmitrov, Aleksandrov, Pavlovsky Posad, Kolomna, Serpukhov, Maloyaroslavets stations), and the heat island’s intensity, which is defined as the temperature difference between the station Balchug and average background values between 1977 and 2016. (**b**). Black dashed lines show linear trends for between 1977 and 2016. UHI is Urban Heat Island.

The Moscow region has faced significant climate changes over the past decades. Regional climate warming is now most pronounced since the 1970s, and is especially pronounced in the summer (Figure 2a,b). Between 1977 and 2016, the average rate of growth of the mean summer temperature (June–August) was 0.6 °C per decade for rural areas [21]. The Moscow megacity forms an intensive urban heat island [22], which manifests itself as a mesoscale temperature anomaly that covers the whole city and even its neighboring regions [21,23]. The urban–rural temperature contrasts could reach up to 13 °C in favorable weather conditions, while the annual mean temperature difference between the city center and rural surroundings is about 2 °C [21,24]. Urban growth in recent decades has resulted in the intensification of the urban heat island (Figure 2b) and caused additional amplification of the climate warming rates for urban areas [21,25].

Hence, the Moscow megacity is a good example of a densely populated urban area, which is affected by pronounced climate changes and by a variability of local climates, and is threatened by vivax malaria outbreaks. This study is based on actual weather observations over the past 40 years. It evaluates the significance of the regional recent climate changes and the heterogeneity of local climates in the Moscow region in terms of climatic favorability for the transmission of vivax malaria.

## 2. Materials and Methods

### 2.1. Study Area

Administratively, the Moscow region consists of two units, which are both plenipotentiary members of the Russian Federation viz. the Moscow city and the Moscow *oblast*’.

**Figure 3:**
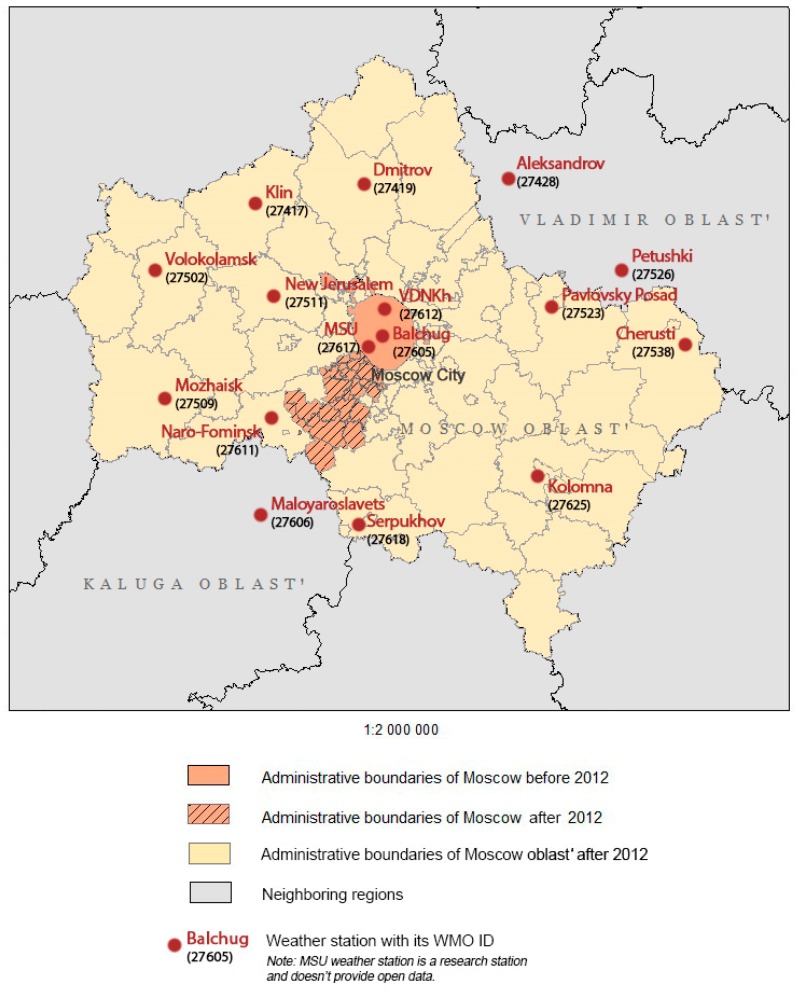
Moscow region geographical position.

The border between them was changed in 2012, but in the text, we refer to the pre-reform status. By the beginning of 2012, the population of the city and the *oblast*’ was 11.6 million and 7.2 million inhabitants, and the areas were 2561.5 sq km and 44.329 sq km, respectively (data of Russian Federal State Statistics service) [26] (Figure 3). The territories of the neighboring regions are also considered.

### 2.2. Meteorological Data

To analyze the change in the degree of favorable climatic conditions for the extrinsic development of the malaria parasite, we used long-term observational data from 16 weather stations, which are operated under the standards of World Meteorological Organization (WMO). Three of them are located in Moscow city, including Balchug station (WMO ID 27605), which is located in a densely built area just in the city center, VDHKh station (WMO ID 27612), which is located in a park area nine km to the north from the city center, and the meteorological observatory of Lomonosov Moscow State University (MSU), which is located in another park area 7.5 km to the southwest from the city center. These three stations represent the climate of the urban environment affected by the urban heat island effect. The annual mean temperature anomaly (deviation from the mean rural value) is 2 °C for Balchug station and about 1 °C for the park stations MSU and VDHKh [24]. According to the recent detailed studies of Moscow climate features [21,23], the temperature observations at Balchug station are representative of the whole central part of the city, and of some especially densely built areas beyond. Most of the other built areas within the city are typically cooler than the city center, but warmer than the urban parks, and, moreover, rural areas. For rural areas, we use data for 13 weather stations that are located in Moscow *oblast*’ or in the neighboring parts of Vladimir *oblast*’ and Kaluga *oblast*’: Volokolamsk (WMO ID 27502) Klin (27417), Dmitrov (27419), Pavlovsky Posad (27523), Cherusti (27538), New Jerusalem (27511), Mozhaisk (27509), Naro-Fominsk (27611), Serpukhov (27618), Kolomna (27625), Maloyaroslavets (27606), Aleksandrov (27428), and Petushki (27526). Listed stations are located in rural areas or at the edges of the small towns, so they represent almost natural environments in different parts of the Moscow region.

The further analysis is based on the time period from 1977 to 2016. It was selected according to the availability of observation data. This time period also allows examining a rather monotonous climate-warming pattern (see Figure 2), which simplifies the analysis of trends.

A database of temperature observations on three-hourly intervals (at zero hours, three hours, six hours, 12 h, 15 h, 18 h, and 21 hours UTC) for the period between 1977 and 2016 was created on the basis of archives of RIHMI-WDC (Russian Institute for Hydrometeorological Information—World Data Center), Central Department for Hydrometeorology and Environmental Monitoring, website “Weather Schedule”, and the meteorological observatory of Moscow State University.

Average daily temperatures (ADT) were calculated based on observations on three-hourly intervals. The missing data ratio did not exceed 5% for all the weather stations considered, and we applied a gap-filling algorithm to restore missing ADT values. This algorithm is based on the method of [27]. Missing values for a given station were filled using the multiple linear regressions between existing ADT values for this station and a number of the nearest stations. Such an approach allows restoring missing values while taking into account the typical local climate features.

In order to quantify the baseline rural conditions, we consider the mean rural ADT, averaging nine rural stations (Klin, Dmitrov, Alexandrov, Pavlovsky Posad, Kolomna, Serpukhov, Maloyaroslavets, Naro-Fominsk, and New Jerusalem).

### 2.3. Quantitative Indicators of Climatic Favorability for Malaria Transmission

As mentioned above, the spread of malaria in temperate areas is limited by the temperature requirements of *Plasmodia* at the extrinsic part of their life cycle. For the development of the sporozoites of *P. vivax* to occur, the ADT should be no lower than +16 °C. If this condition is met, the ADTs excesses over the threshold of 14.5 °C are summed up, and when the sum reaches 105 °C, this indicates that the sporogony is over [28]. If ADTs fall below 16 °C, this particular day is excluded. In this case, sporogony is interrupted, but it can resume if the break is shorter than a week. It is important to note that when the ADT is close to the lower threshold, the maturation of sporozoites goes so slowly that the time required surpasses the life span of mosquitoes, and the possibility of transmission becomes negligible. At higher ADTs, the development of sporozoites continue to accelerate up to about 30 °C. Temperatures above 30 °C are deleterious for both parasites and mosquitoes.

The structure of each malaria season over the 40-year period was evaluated according to [29]. This method is recommended by the WHO [30], and is optimal for temperate areas where malaria transmission is possible for only one or a few months per year. At the same time, this method is of little use for other areas such as tropical climates, where ADTs always or almost always permit the development of sporozoites, and where the dynamics of malaria are often defined by rainfall. It calls for other methods of forecasting, such as for example, the Gradient Model Risk Index, which is based on a detailed analysis of the environmental conditions for the vector (the dynamics of vector populations, vector’s ability to transmit the infection), and, occasionally, taking into account human cases [31,32,33,34,35].

**Figure 4:**
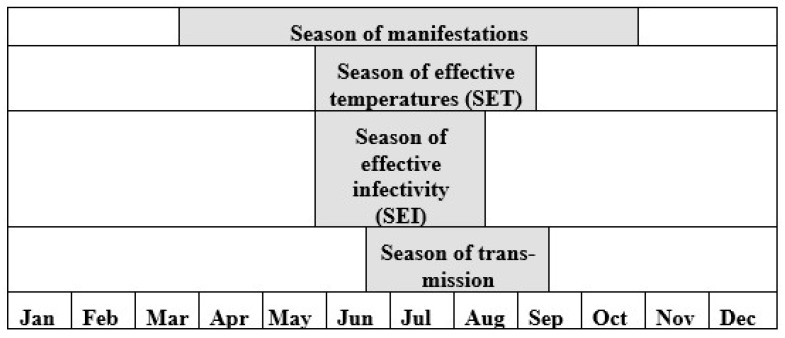
Interrelation of the elements of malaria season (e.g., in Central Russia), adopted from [30].

The following elements of the malaria season have been identified and calculated (Figure 4):

- Season of manifestations: the period of the year during which most malaria manifestations start. It begins before the activity of mosquitoes, since a significant proportion of vivax malaria cases occurs nine to 12 months after the inoculation due to the long latency period [36];
- Season of effective temperatures (SET): the period of the year during which ADTs consistently keep above the parasite development threshold (+16 °C for *P. vivax*);
- Season of effective infectiveness of mosquitoes (SEI): the period during which a mosquito infected on a person can generate mature sporozoites. It starts at the same moment as SET, but stops earlier; this is because mosquitoes that contracted parasites nearer to the end of the SEI do not have enough time at a favorable temperature to generate fully developed parasites (the infection of mosquitoes is not effective in this case).
- Malaria transmission season: the period during which mosquitoes with mature sporozoites can infect humans. It starts later than the SEI by the lag equal to the duration of the period for development of a single generation of sporozoites, which may take three to four weeks at the beginning of the warm period.

The timing of events of the malaria season gives a useful basis for comparisons and forecast for malaria control in those situations in which the transmission is deemed possible. However, this approach does not differentiate between situations in which the above calculations indicate that the transmission seems impossible. In fact, when the calculations predict the impossibility of the transmission, this may be accounted for by different circumstances. There would be occasions when the probability of the maturation of sporozoites is definitively zero (say, if ADT does not reach 16° all over the period). On the other hand, there would be occasions when the sums of temperatures come very near to the required values. On such occasions, due to the spatial variation of microclimatic conditions, in which mosquitoes dwell during their incessant journeys between the feeding and breeding places (which may be only coarsely appreciated by the temperatures at the meteorological stations), vectors may occasionally find themselves in warmer conditions than those predicted by the records. As a result, the maturation of sporozoites may occasionally take place, despite the prediction.

In order to take into account those marginal situations when the probability of the transmission is slightly above zero, we propose the following integral index of favorability of thermal conditions, which is based on the assumptions that in order to complete one infection turnover (from the ingestion of gametocytes by a mosquito to the mosquito becoming infective), no less than a sum of effective temperatures (ET) of 105 °C is required. When it happens, this may generate a small number of secondary cases, but for the transmission to become established, another round of maturation of sporozoites would be required. In other words, no less than 210 °C would be needed (that is notwithstanding that at least additional 10 days would be needed for an infected person to generate gametocytes, but since this span is temperature-independent, it cannot be absorbed into the model).

Thus, let:

K_f_ be the index of favorability of thermal conditions;

S_obs_ be the observed sum of ET;

S_obs_ be the observed sum of ET;

S_min_ be the required sum of ET for a particular species of malaria: 210 °C in case of *P. vivax*

K_f_ = S_obs_/S_min_ (1)

The interpretation of particular values of the index is shown in Table 1.

Finally, temporal trends and their significance were analyzed for individual malaria seasons. For each station, the slope coefficient of the linear trend k, the coefficient of determination *R*^2^, and the confidence level p at which the trend would be statistically significant by the Student’s criterion have been calculated.

## 3. Results

Over the past 40 years (1977–2016), all three indicators of climatic suitability in relation to *P. vivax* (*viz*., the sums of effective temperatures, the duration of the season of effective infectivity, and the index of favorability (K_f_)) continued to rise.

### 3.1. Sums of Effective Temperatures (ET)

**Figure 5:**
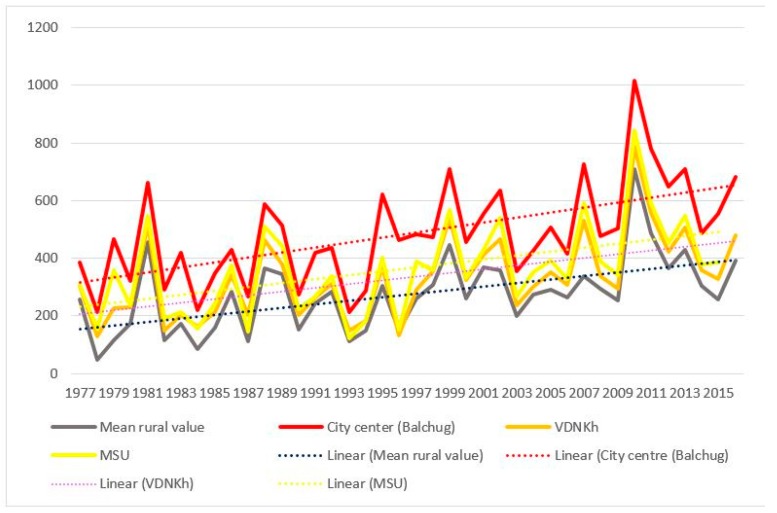
Sums of effective temperatures accumulated in Moscow city and suburban areas during the years 1977 to 2016 (dashed lines show linear trends).

The dynamics of the sums of ETs accumulated is presented in Figure 5.

The following salient points should be noted:

1. Sums of ETs for the city center remain well above the average rural values every year, whereas the values for the park zones stay in between the two.
2. For the city center, temperatures allowed the full development of at least one generation of sporozoites every year. For individual rural stations, there were years when the temperatures remained below the threshold of 105 °C, i.e., the temperatures were insufficient to ensure the transmission of *P. vivax*. The average rural values moved below the threshold only twice, in 1978 and in 1984 (before 1977 not shown on the graph); there were regular cases of years when temperatures remained prohibitive to the development of the parasite.
3. There was a growing trend of sums of ETs, especially after 1997. For average rural values, the coefficient of the linear trend *k* equals 65 °C/10 years, which is statistically significant (*R*^2^ = 0.31, *p* < 0.01). This trend is further emphasized by the sums of ETs reaching below the threshold only once after 1997, in the year 2015, and in one place, i.e., in Volokolamsk (which is known as a “cold” station, where this indicator is always lower than in the other points).
4. The growth rate of the sums of ET for the urban stations exceeded the growth rate in the rural stations: the trend slope coefficient and its significance for these two stations was greater than for the rural ones, revealing the intensification of the urban heat island.

**Figure 6:**
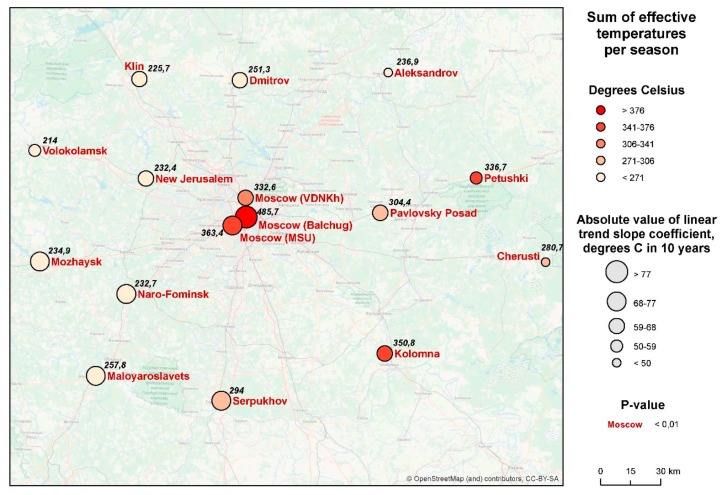
Spatial distribution of changes in the sum of effective temperatures in the Moscow region in 1977–2016 (figures for each station indicate the average amount of effective temperature, or ET, for the period).

The geographical distribution of the phenomenon is shown in Figure 6. Here, the average values of the sums of ET are shown by shadings, and the tempo of their increase during the 40-year period is shown by the size of the circles (a similar approach is applied further for the other two indicators in figures 8 and 10). A marked spatial heterogeneity may be seen, with the values being consistently higher in Moscow city and in the eastern parts of the region. For Moscow city, this may be explained by the effect of the urban heat island; for the east of Moscow region, by the rise in the continentality of the climate: west to east, the winters grow colder, but summers are hotter. At the same time, the rate of change of the indicator is more significant in the central and western parts of the region.

### 3.2. Duration of the Season of Effective Infectivity (SEI)

**Figure 7:**
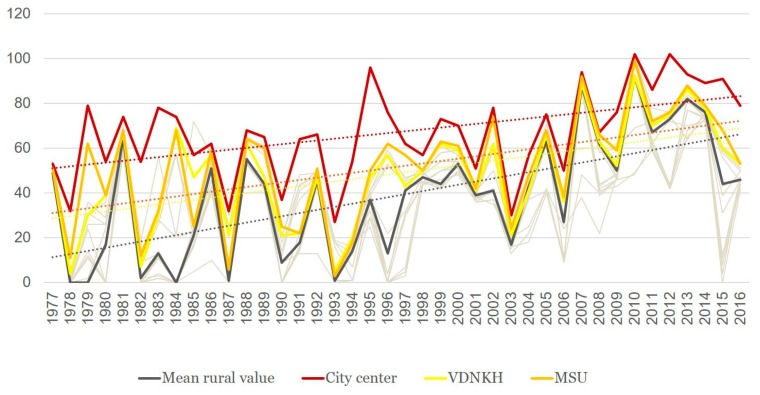
Change in the duration of season of effective infectivity (SEI) at urban and suburban stations, in comparison with the average background conditions in the Moscow region in 1977–2016 (dashed lines show linear trends, thin gray lines indicate the rest of the analyzed stations).

Over the period of 40 years, the duration of the SEI displays a similar tendency of rise (Figure 7). This is true for all the points under consideration, but there is a marked variation between them. During the same given year, the difference may be up to 37 days. The SEI is longer in Moscow city than at the periphery, and longer in the eastern and southern parts of the Moscow *oblast*’ than elsewhere. At the same time, the rate of increase in the duration of the season is more significant in its western and southwestern parts.

**Figure 8:**
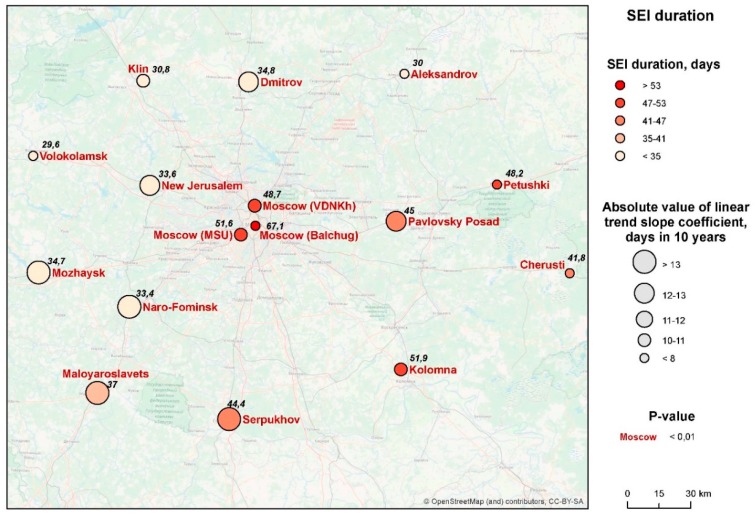
Spatial distribution of changes in the duration of the SEI in the Moscow region (the numbers for each station indicate the average duration of the SEI for the period).

Balchug station in the city center has consistently demonstrated the longest SEI duration compared with all other stations (67.1 days on the average for the whole period of 40 years). However, in contrast to the sum of ET, the tendencies to increase the duration of the season in the city center are less pronounced than those in the rural areas (*k* = 8.2 days/10 years, which is less than in any of the stations considered; *R*^2^ = 0.27) (for rural areas *k* = 14.1 days/10 years; *R*^2^ = 0.40) (Figure 8). It is probable that a further increase in the duration of the SEI becomes impossible, since the SEI occupies most of the warm period of the year anyway, due to the influence of the urban heat island. In other words, the longer the SEI, the less is the sensitivity of malaria to further climate warming. This pattern, though with some exceptions, is also shown by rural stations.

There is a tendency for the entire Moscow region for a shift of the date of the beginning of the SEI to an earlier date, but the significance of these trends is small. In the rural areas, on the average, this date advanced by *k* = −6 days/10 years (*R*^2^ = 0.16, *p* = 0.013). In other words, the average date of the beginning of the SET was the first week of June at the beginning of the 40-year observation period, and the last week of May at the end. Within the limits of Moscow city, the SEI always starts earlier than in the rural areas; the southern parts of Moscow *oblast’* are also marked by earlier dates. However, the spatial variability is not so great, with the maximum differences within the whole of the area not exceeding nine days.

The date of the end of the SEI for the rural average was the first half of July at the beginning of the 40-year period, and the end of July/the beginning of August at its end. The trends of the SEI ending later over the years were more pronounced than the trends of SEI commencing earlier. For the rural average, *k* = 7 days/10 years, *R*^2^ = 0.26, *p* < 0.01.

The spatial variability of the dates of the end of the SEI largely repeats the patterns of the change in its beginning and its total duration: the season ends later in the southern parts of the region and in Moscow city. In other words, warmer autumns are the main contributor to the increase in the duration of the malaria season.

### 3.3. Index of Favorability (K_f_)

**Figure 9:**
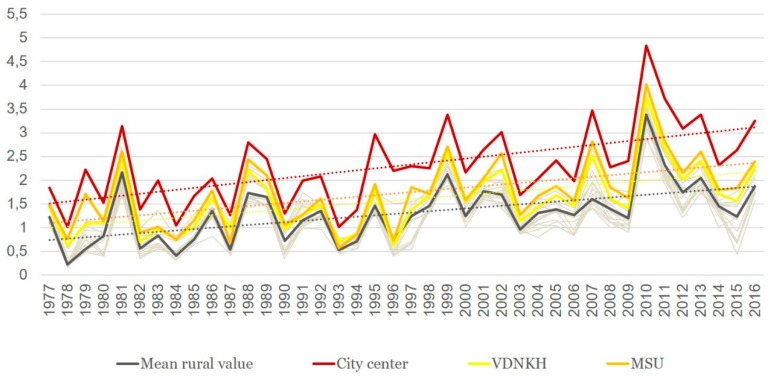
Changes in the K_f_ (index of favorability) in the Moscow region between 1977 and 2016 (dashed lines show linear trends, and thin gray lines indicate the rest of the analyzed stations).

The change in the degree of favorability of the thermal regime is better demonstrated by spatio-temporal analysis of the integrated index K_f_. The graph of K_f_. (Figure 9) clearly confirms the conclusions made earlier for the two other indicators.

**Figure 10:**
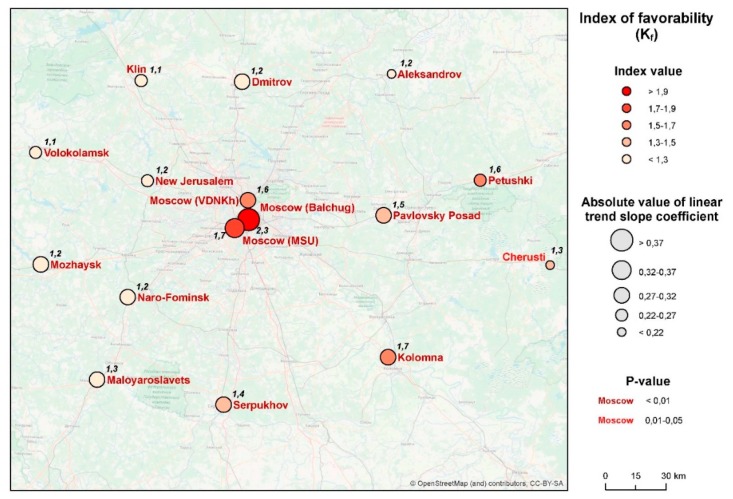
Spatial distribution of changes in the K_f_ (index of favorability).

First, the climate of the Moscow city (especially of its center) is much more favorable for malaria than that of the periphery. Second, there is a significant (*p* < 0.01) trend of increase of K_f_, but its speed is very variable (Figure 10).

**Figure 11:**
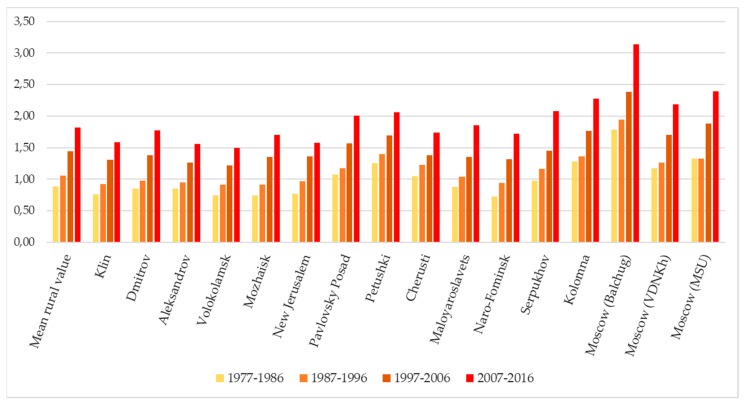
Changes in the degree of favorability of thermal regime for the development of the vivax malaria parasite in the Moscow region over the 10-year periods between 1977 and 2016.

When analyzing data by the 10-year periods, there is a noticeable progressive increase in the degree of favorability of the climate (Figure 11). Whereas the change was insignificant in the period from 1977 to 1996, it is more noticeable during the period from 1997 to 2016. At the same time, for the western and northern (less continental) parts of the region, the greatest changes related to between 1997 and 2006, especially in the Klin, Mozhaisk, and New Jerusalem stations, while for the rest of the territory, the shift was more pronounced during the period from 2007 to 2016. Finally, due to the intensification of the heat island, the growth trend of the index was most pronounced in the city center, especially during the last decade.

These changes are also reflected in a considerable growth trend of the K_f_ index for all the stations in the Moscow region, as well as its variation within the region (Figure 11).

## 4. Discussion

A great number of scenarios depicting the changes inflicted by global warming have been published during the past three decades. By and large, they predict that the global warming *ipso facto* would result in a deterioration of the malaria situation.

However, these works did not always address the following:

The two main species of human malaria are rather distant ecologically: that is why we consider only one species, *P. vivax*;

The limiting factors of malaria are not the same in the tropics and in the temperate areas (rainfall versus temperature, respectively): that is why we examine a particular case of environment of Central Russia;

In temperate areas, malaria is limited by the requirements of the parasite, and not those of the vector: that is why we do not consider the vector.

There has been a considerable number of studies on the influence of climatic change on the future malaria situation. They are based on various prognostic scenarios that appear more or less realistic. For instance, Lindsay et al. [37] provided risk maps indicating the territory that was climatically suitable for vivax malaria distribution, which nowadays includes central and southern England. This territory will probably increase in extent in the future, reaching southwestern Scotland by 2030. According to this scenario, in some places in southern England, the duration of malaria transmission season may exceed three to four months.

Caminade et al. [34] forecasted an overall global net increase in climate suitability for malaria transmission and an increase in the population at risk from the 2050s to the 2080s. They believed that, according to some scenarios, malaria epidemic belt may shift northward over central–northern Europe, Russia, northern Asia, and northern America. It is specially highlighted in their work that the results of such prognoses are quite uncertain due to differences in the initial malaria impact models that were selected for the prognosis, especially over the periphery of the malaria distribution range. Unfortunately, in this paper the authors regarded malaria as a single entity, ignoring the differences in the temperature requirements of particular parasite species, which may have invalidated their prognosis.

On the contrary, some forecasters doubted a climate-driven increase of the area of distribution of malaria. For example, Semenov et al. [38] suggested that climate change is unlikely to lead to considerable changes in the territory suitable for malaria transmission in the Russian Federation.

There was an assumption supported by many researchers that while climatic factors may favor autochthonous transmission by increasing vector densities and accelerating the parasite development, the re-emergence of malaria in Europe (e.g. temperate regions) would hardly occur due to other counteractive factors, primarily the socioeconomic changes in land-use and agriculture practices, and effective functioning of the public health system [37,39,40,41].

We feel that the climatic prerequisites should be considered separately from the other prerequisites e.g. those related to socioeconomic factors and public health. The availability of climatic prerequisite is a *conditio sine qua non*, but their presence does not automatically mean that the area is at malaria risk. That is why we limit ourselves by analyzing climatic prerequisites only.

The real danger of occasional outbreaks following the importation of malaria was demonstrated in a recent episode of vivax malaria transmission in Greece [42]. Climate change may act as a trigger factor for malaria reintroduction in countries with insufficient malaria surveillance, uncontrolled migrations, and a lack of malaria awareness among health professionals and travelers. Van Lieshout et al. [43] considered Russia among the “countries where malaria remains at a low level despite poor control efforts” with previously “effective control program [having] declined in recent years”. In the authors’ view, such countries may be migrating toward a high malaria status, and it is unlikely that they will have “the structural or economic capacity to cope with any increases in malaria that climate change will bring”. In this regard, it can be concluded that the authors consider climate change to be an important cause of the deterioration of malaria status. However, we feel that malaria control in Russia, in fact, has been quite reasonable in the past, and has not changed much in strategy and scope since the period of massive importation of malaria from Afghanistan in the 1980s. This “low level” was an achievement by itself in view of an unprecedented influx of infected individuals from Tajikistan and Azerbaijan experiencing severe epidemics at that time, which was aggravated by climatic change.

In the case of Russia, the reintroduction of malaria manifested itself in the most aggressive form in the Moscow region [44]. Historically, thermal conditions for the transmission of tertian malaria have been, in general, moderately favorable in Moscow region, although there were years when the transmission was impossible due to cold summers. The infection was endemic in this area, including the megapolis (which means that its transmission was observed practically annually) right until its elimination in the mid-1950s [45,46,47,48].

Thermal conditions started to change in the region since the beginning of the 1980s. In 1981, thermal conditions for malaria were better than ever. Since then, a rising trend has been observed (Figure 8). This is demonstrated by the SEI becoming longer. This prolongation is due, to some extent, to an earlier beginning of SEI, but is mostly due to its delayed ending. The index of favorability K_f_ that we proposed is also on the rise, and seems to be a reliable indicator of the quality of weather in relation to malaria.

The trends of the three indicators is generally consistent with the general warming of the Moscow region, which has accelerated since the early 1990s, with an increase in summer temperature and an intensification of the summer urban heat island [21].

These indicators, as well as the speed of their change, demonstrated a spatial heterogeneity. The climatic conditions of Moscow city and, especially, of its central part are much more favorable for the development of the malaria parasite than the conditions in rural areas. This is probably due to the influence of the urban heat island. However, better thermal conditions might be counterbalanced by the deterioration of the conditions for mosquito proliferation, due to the urbanization and pollution.

For rural areas, conditions are more favorable in relation to malaria in the south than in the north of the *oblast*’, which is due to the general warming of the climate in southern direction. At the same time, the conditions are more favorable in the east than in the west, due to a more continental climate in the east. As a result of the interaction of these factors, the thermal conditions for malaria are more favorable in the city of Moscow and least favorable in the western and northern parts of the Moscow *oblast*’.

Besides the increase in the favorability of climatic conditions for malaria parasites, warming may also affect the vector populations. The phenology of urban mosquitoes demonstrates a shift to earlier terms comparing rural and suburban areas, and this was associated with an urban heat island effect, which raised urban air and water temperatures [49]. However, the increase of climatic favorability for malaria vectors *per se* doesn’t lead to any deterioration of the malaria situation. This is because malaria transmission is limited by the thermal requirements of the parasite, which are narrower than those of the vector. This is often ignored by some researchers who have equated the climate-driven vectors’ range transformations and the changes in potential malaria range [50,51].

It is generally believed that the causes of decline of malaria in Europe during the first half of the 20th century were related mostly to socioeconomic improvements, whereas the changes in climate and land use played a less important role [52]. However, it seems that the climatic changes may play a more significant role in malaria resurgence nowadays.

It is true that the causes for the resurgence of malaria transmission in the Moscow region after about two decades of its total absence obviously point to the widespread epidemics in Central Asia and in Azerbaijan, on the background of intensive labor migration. However, this process was likely to have been modulated by climatic change.

As it was mentioned above, the transmission of vivax malaria in Moscow city and *oblast*’ lasted from 1999 to 2008. During this period, there were four years with unusually hot summers, *viz*. 1999, 2002, 2007, and 2010. During these warm summers, important epidemiological events took place. In 1999, malaria transmission restarted, and in 2002, it reached its peak. At the same time, nothing remarkable happened during the hot summers of 2007 and 2010. This is probably due to a radical improvement of the situation in Central Asia and Azerbaijan, and the cessation of the importation of malaria in Russia [48].

Climate change may act as a trigger factor for malaria reintroduction in countries with insufficient malaria surveillance, uncontrolled population movement, and lack of malaria awareness among health professionals and travelers. Despite the predominant perception that urbanization would rather decline the possibilities of malaria transmission in big cities mostly due to socioeconomic improvements [52,53,54], the lessons being learned from the recent re-emergence of vivax malaria in Moscow region, where the degree of antimalaria preparedness was traditionally high), demonstrate that malaria transmission can restart due to some triggering factors, such as climate change.

## 5. Conclusions

In this research, we have demonstrated the patterns of spatial variability and long-term dynamics of the climatic favorability to malaria in Moscow megacity and its surroundings. Due to the climate warming, the climate of the region has become significantly more conducive to a new resurgence of malaria. This mandates the continuation of antimalaria preventive activities, despite the interruption of malaria transmission that had been achieved. The most favorable climatic conditions were found within Moscow megacity, especially in the city center, which is warmer than the rural surroundings due to the urban heat island effect. In the central part of the city, the season of effective infectiveness is longer by 25 days, and the integral index of climate favorability is higher by 85% in comparison to mean values over the rural surroundings. Moreover, the observed intensification of the urban heat island additionally amplifies the rates of summer climate warming and the growth of favorability for malaria transmission. Such favorable conditions seem to be mostly counterbalanced by the hostility of the highly urbanized environment toward the anopheline vector. The presented results show the importance of taking the local climatic features of urban environment into consideration in malaria studies, as well as monitoring antimalaria preventive activities.

## Figures and Tables

**Table 1:** The extent of favorability of meteorological conditions for autochthonous transmission of malaria.

Index of Favorability of Thermal Conditions (K_f_)	Transmission	Epidemiological Outcomes when an Imported Case Appears
<0.5	No transmission	Occurrence of secondary (introduced) cases is impossible.
0.5–1	Unfavorable (shortened) season, a sporadic transmission	Occurrence of a few introduced cases is possible; however, these would not produce a new generation of tertiary cases.
≥1	Favorable season, an established transmission	In addition to a few introduced cases at the beginning of the season, a second generation of tertiary cases may appear (indigenous cases), thus producing an epidemic.
